# Neoadjuvant Chemotherapy Induces Expression Levels of Breast Cancer Resistance Protein That Predict Disease-Free Survival in Breast Cancer

**DOI:** 10.1371/journal.pone.0062766

**Published:** 2013-05-02

**Authors:** Baek Kim, Hiba Fatayer, Andrew M. Hanby, Kieran Horgan, Sarah L. Perry, Elizabeth M.A. Valleley, Eldo T. Verghese, Bethany J. Williams, James L. Thorne, Thomas A. Hughes

**Affiliations:** 1 Leeds Institute of Molecular Medicine, University of Leeds, Leeds, United Kingdom; 2 Department of Breast Surgery, Leeds General Infirmary, Leeds, United Kingdom; 3 Department of Histopathology, St. James’s University Hospital, Leeds, United Kingdom; Johns Hopkins University, United States of America

## Abstract

Three main xenobiotic efflux pumps have been implicated in modulating breast cancer chemotherapy responses. These are P-glycoprotein (Pgp), Multidrug Resistance-associated Protein 1 (MRP1), and Breast Cancer Resistance Protein (BCRP). We investigated expression of these proteins in breast cancers before and after neoadjuvant chemotherapy (NAC) to determine whether their levels define response to NAC or subsequent survival. Formalin-fixed paraffin-embedded tissues were collected representing matched pairs of core biopsy (pre-NAC) and surgical specimen (post-NAC) from 45 patients with invasive ductal carcinomas. NAC regimes were anthracyclines +/− taxanes. Immunohistochemistry was performed for Pgp, MRP1 and BCRP and expression was quantified objectively using computer-aided scoring. Pgp and MRP1 were significantly up-regulated after exposure to NAC (Wilcoxon signed-rank p = 0.0024 and p<0.0001), while BCRP showed more variation in response to NAC, with frequent up- (59% of cases) and down-regulation (41%) contributing to a lack of significant difference overall. Pre-NAC expression of all markers, and post-NAC expression of Pgp and MRP1 did not correlate with NAC response or with disease-free survival (DFS). Post-NAC expression of BCRP did not correlate with NAC response, but correlated significantly with DFS (Log rank p = 0.007), with longer DFS in patients with low post-NAC BCRP expression. In multivariate Cox regression analyses, post-NAC BCRP expression levels proved to predict DFS independently of standard prognostic factors, with high expression associated with a hazard ratio of 4.04 (95% confidence interval 1.3–12.2; p = 0.013). We conclude that NAC-induced expression levels of BCRP predict survival after NAC for breast cancer, while Pgp and MRP1 expression have little predictive value.

## Introduction

Xenobiotic transporters are transmembrane efflux pumps that have protective physiological roles by removing potentially harmful molecules from the intracellular environment. These transporters can also cause efflux of common chemotherapeutic agents and accordingly their activities have been implicated as mechanisms for therapy resistance in many cancer types. There are more than 30 individual human genes for xenobiotic transporters, but three specific family members have been most frequently implicated as modifiers of chemotherapy response in breast cancers [1]. These are P-glycoprotein (Pgp; encoded by the ABCB1 gene), Multidrug Resistance-associated Protein 1 (MRP1; encoded by the ABCC1 gene), and Breast Cancer Resistance Protein (BCRP; encoded by the ABCG2 gene).

Pgp has a broad range of substrates and exports many classes of chemotherapeutics including anthracyclines and taxanes [2]. Many studies have investigated Pgp expression levels and their prognostic impact in breast cancer [3], but use of a variety of assay methods has lead to widely differing detection rates and therefore conflicting conclusions [4]; some find Pgp expression to be associated with poor prognoses [5], while others do not [6]. There is, however, broad consensus that Pgp expression can be induced by neoadjuvant chemotherapy [7], suggesting that Pgp may contribute to acquired chemotherapy resistance in some cases. Compared to Pgp, the other two transporters have been studied relatively little. MRP1 has a similar range of substrates to Pgp, however it appears to be unable to export taxanes [8]. MRP1 is expressed in the vast majority of breast cancers as well as in some normal breast tissues, and high expression within breast tumours has generally been found to correlate with poor prognosis [4]. Like Pgp, MRP1 expression is reportedly induced by neoadjuvant chemotherapy, although only pre-treatment expression and not post-treatment expression correlated with prognosis therefore the clinical relevance of induction is not clear [7]. BCRP can export a range of substrates, although it may not act upon taxanes [8]. The action of BCRP on anthracyclines is influenced, at least in cell lines, by the presence of somatic mutations within the gene, with some mutants acting on them effectively, while the wild-type protein may act poorly [9]. Although these mutations have never been identified in clinical cancers, some authors have found BCRP to correlate with response to anthracycline-based therapy [10], so the influence of BCRP on anthracyclines in a clinical setting remains unclear [11]. A number of coding single nucleotide polymorphisms also influence BCRP function and substrate specificity [12], adding further confusion. Surprisingly given its name, relatively little is known about the expression or prognostic value of BCRP in clinical breast cancers, although over-expression has been associated with drug-resistance in many *in vitro* studies [13], and higher expression has been associated with more advanced disease [14].

Neoadjuvant chemotherapy (NAC) is increasingly used in breast cancer treatment to downstage tumours, to allow more frequent use of breast conserving surgery as an alternative to mastectomy [15], and to improve breast cancer survival. Response to NAC is typically monitored by non-invasive magnetic resonance imaging (MRI) during therapy and by analysis of resection tissue after surgery [16]. Reported response rates vary substantially with much of this variation associated with differences in patient cohorts, NAC regimen, and response measurement and reporting [17]. Critically, patients who have favourable responses to NAC, as determined by MRI or pathology, are thought to have improved disease-free survival [18]. However, despite much research, little insight has been gained into molecular differences between tumours that respond well to NAC and those that respond poorly. Therefore, no molecular markers are in use to predict either NAC responses or how responses might reflect survival gains, although very recently some potential markers have been reported, such as TMSB15A [19] or CD133 [20]. In an effort to identify predictive markers, we investigated expression of Pgp, MRP1, and BCRP in a cohort of NAC-treated breast cancer patients. We examined expression both pre-treatment and post-treatment with a view to testing whether either predicted NAC response or survival.

## Methods

### Ethical Issues, Patient Cohort and Patient Samples

Ethical approval was obtained from Leeds (East) Research Ethics Committee (reference 06/Q1206/180); written consent was taken from patients as approved by the committee. The patient cohort comprised patients treated with neoadjuvant chemotherapy (NAC) for primary breast cancer at Leeds Teaching Hospitals NHS Trust from 2005–2009. Further selection criteria were minimum 3 years clinical follow-up after NAC, grade 2 or 3 invasive ductal carcinoma, NAC treatment with cyclophosphamide and epirubicin with/without taxanes, and post-operative radiotherapy (n = 45). Relevant clinical and pathological parameters are described in Table 1. Archival formalin-fixed paraffin-embedded (FFPE) breast tissue blocks representing both core biopsies (pre-NAC) and matched resection tissue (post-NAC) were collected. Eight patients were reported as having achieved complete pathological responses. Tissue blocks representing resection tissue from these patients were step-sectioned at depths of 100 µm; sections from each level were stained with the anti-pan cytokeratin AE1/3 antibody (M 3515; Dako, Glostrup, Denmark). This revealed tumour cells in two cases.

**Table 1:** Clinical and pathological features of the patients.

Characteristic	Categories	No. of patients (%)n = 45
Age	<45	21 (47)
	>45	24 (53)
Grade (pre-NAC)	2	15 (33.3)
	3	30 (66.7)
Pre-NAC stage (based on MRI)	T2	26 (57.8)
	T3	16 (35.6)
	T4	3 (6.6)
Post-NAC stage (based on resection pathology)	T0	6 (13.3)
	T1	14 (31.1)
	T2	17 (37.8)
	T3	6 (13.3)
	T4	2 (4.5)
Tumour size change (initial size: MRI; final size: resection pathology)	increase	8 (17.8)
	decrease	31 (68.9)
	not assessable (pCR: no residual tumour cells identified)	6 (13.3)
MRI response	minimal	9 (20)
	partial	30 (66.7)
	complete	6 (13.3)
NAC regimen	epirubicin+cyclophosphamide (EC)	13 (28.9)
	EC+taxanes	32 (71.1)
Lymphovascular invasion	positive	17 (37.8)
Axillary metastasis	positive	21 (46.7)
Estrogen receptor	positive	26 (57.8)
Her2	positive	9 (20)
Surgery	breast conserving	17 (37.8)
	mastectomy	28 (62.2)
Follow up	median: 4.5 years (range 3–8.8 years)	
Recurrence		17 (37.8)
Death		10 (22.2)

### Immunohistochemistry

Tissues were sectioned at 5 µm onto SuperFrost Plus slides (Menzel-Glaser, Braunschweig, Germany). Matched biopsy and tumour containing resection samples were placed on the same single slide therefore subsequent staining and analysis conditions for each pair were identical and relative expression between matched samples were directly comparable (39 cases). A further 6 core biopsies were sectioned/processed without matched resection tissue since resections lacked identifiable tumour cells. Slides were air dried and incubated (overnight, 37°C). The following antibodies were used: UIC2 for Pgp (sc-73354; Santa Cruz Biotech., Santa Cruz, USA), QCRL1 for MRP1 (sc-18835; Santa Cruz Biotech., Santa Cruz, USA), and BXP21 for BCRP (ab3380; Abcam, Cambridge, UK). All three antibodies have been used widely for immunohistochemistry on breast tissue [21]–[23], and their specificities have been validated previously using Western blotting on breast cell lines [24], [25]. Sections were dewaxed with xylene and rehydrated with ethanol. MRP1 epitopes were retrieved by heat (900W microwave, 10 min) in 10 mM citric acid buffer (pH 6.0); epitope retrieval was not performed for other antigens. Endogenous peroxidase activity was blocked using 0.3% H_2_O_2_ (10 min). Non-specific binding activity was blocked using casein solution diluted 10-fold in tris-buffered saline (SP5020; Vector Labs, Burlingame, USA) for anti-Pgp only (20 min). Slides were incubated in antibody diluent solution (Invitrogen, Paisley, UK) with primary antibodies (anti-Pgp, 1∶2000, 1 h, room temperature; anti-MRP1, 1∶50, 1 h, room temperature; anti-BCRP, 1∶50, 16 h, 4°C) and were washed in tris-buffered saline. Staining was visualised using Envision reagents (Dako, Gostrup, Denmark). After washing in tris-buffered saline, sections were stained in Mayer’s haematoxylin and Scott’s tap water substitute. Sections were dehydrated and mounted in DPX (Fluka, Gillingham, UK). Negative controls were performed by omitting primary antibodies. Positive controls were performed using breast tumour sections that had showed strong staining for each protein during antibody optimization. Staining was optimized and assessed under guidance of breast histopathologists (AMH and ETV). Sections were digitally scanned using Scanscope XT at 20× magnification and were observed and analysed using Imagescope (Aperio, Vista, USA).

### Histopathological Analysis

Positive (brown) staining was noted in tumour cells only and was quantified with weighted histoscores [26] using an automated protocol. First, our automated protocol was validated. Manual histoscores were given for both the core biopsy and resection components of 30 stained slides (10 for each antigen) by two independent observers (BK and BJW, a histopathologist). The observers counted and semi-quantitatively assessed staining of >100 tumour cells in the same random high power fields. Histoscores (of 0–300) were (1×% of tumour cells weakly stained)+(2×% moderately stained)+(3×% strongly stained). The inter-observer intraclass correlation coefficient (ICC) for these independent manual scores was 0.87 (0.81–1 can be regarded as ‘almost perfect agreement’ [27]). We then used Imagescope (http://tmalab.jhmi.edu/aperiou/userguides/Positive_Pixel.pdf) to compute histoscores for the same fields. Tumour epithelial regions were manually marked on digital images and brown colour within these regions was quantified using the positive pixel count algorithm in three intensity ranges to ape manual scoring (counts of <100 defined as weakly positive, 100 to <175 as moderate, and > = 175 as strong). Pixels that were not counted as brown by software were defined as negative. Percentages of total pixels categorized into each intensity band were used to determine automated histoscores: (1×% weakly positive pixels within epithelial region)+(2×% moderate)+(3×% strong). Inter-observer ICCs between manual scorers and Imagescope were 0.83 and 0.82. After this validation, scoring was performed on the whole cohort by manually marking all tumour epithelial regions throughout sections and using Imagescope to compute histoscores. Statistical analyses were performed using SPSS v16.0 (SPSS, Chicago, USA) with tests described in the text; p values < = 0.05 were considered to indicate significance, except for in Tables S1, S2 and S3 where the more stringent threshold of 0.01 was used.

## Results

### Pgp and MRP1 are Up-regulated after NAC while BCRP Responds Variably

**Figure 1:**
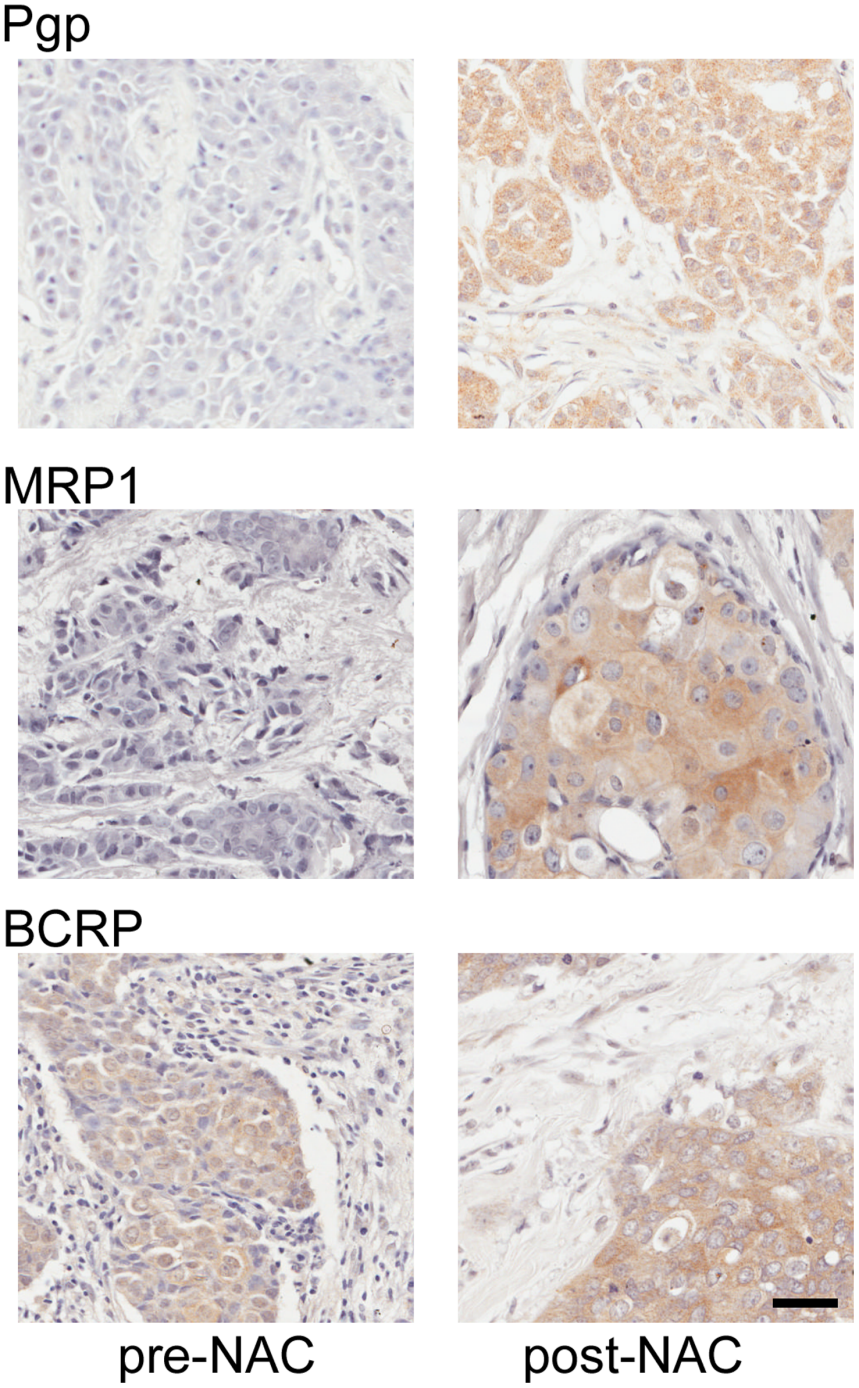
Representative staining patterns for Pgp, MRP1 and BCRP in matched breast tumour tissues pre- and post-neoadjuvant chemotherapy. Individual expression scores for each tissue shown, using a semi-automated scoring system, were: Pgp pre, 10.6; Pgp post, 117.9; MRP1 pre, 3.2; MRP1 post, 55; BCRP pre, 64.5; BCRP post, 89.7. Scale bar: 40 µm.

**Figure 2:**
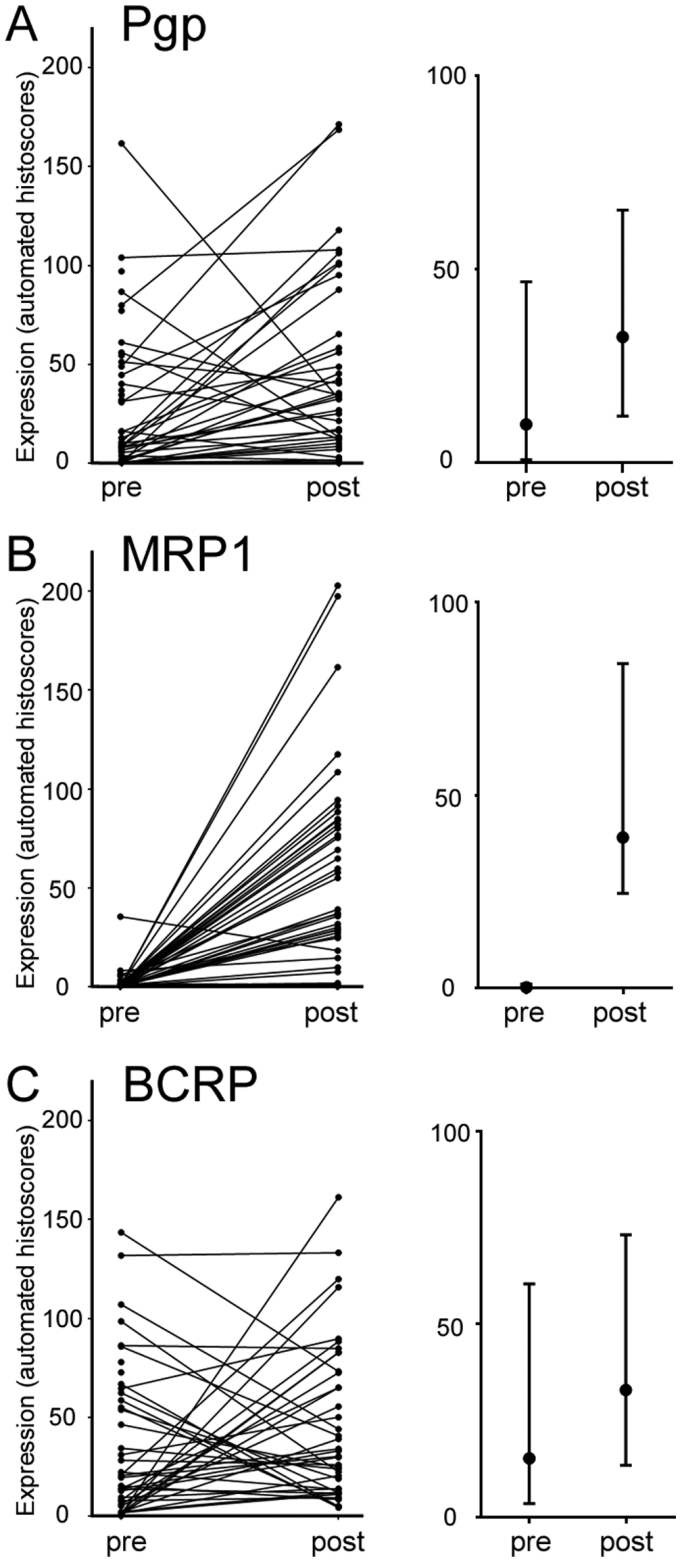
Expression levels of Pgp (A), MRP1 (B) and BCRP (C) in matched breast tumour tissues pre- and post-neoadjuvant chemotherapy (NAC). Left panels show levels in each individual sample with lines linking matched samples (Note: 6 pre-NAC samples are unmatched since these tumours achieved complete pathological responses). Right panels show the median value (central marker) with interquartile range (bars).

We examined expression levels of Pgp, MRP1 and BCRP in the tumours of 45 breast patients treated with NAC. Clinical and pathological features of this cohort are shown in Table 1. In 39 cases, we examined expression both pre-NAC, using diagnostic biopsies, and post-NAC, using surgical resections. Representative staining patterns for each antigen in pre- and post-NAC tissues are shown in Fig. 1. All three markers showed epithelial cell-specific staining that was mainly cytoplasmic, with some accentuation at plasma membranes. Expression of markers within tumour cells was quantified objectively using an automated scoring system. Automated scores were from 0 (no positive staining) to 300 (strongly positive throughout the epithelial cell area), although such a high score was not given in any case; the highest actual score was 203. The scores and their distributions are shown in Fig. 2. Expression of Pgp and BCRP in both pre- and post-NAC samples varied widely from barely detectable to scores of greater than 150 (Fig. 2A, 2C). MRP1 expression, however, was very low in almost all pre-NAC samples, while expression post-NAC was variable, ranging from low to a score of over 200 (Fig. 2B). Scores were compared pre- and post-NAC when matched sections were available. Scores were greater post-NAC than in the matched pre-NAC sample for Pgp in 29/39 cases (74%) and for MRP1 in 36/39 cases (92%), indicating frequent increases in expression in response to NAC. This was reflected in significant differences between pre- and post-NAC scores (Wilcoxon signed-rank tests: Pgp, p = 0.0024; MRP1, p<0.0001). By contrast, BCRP up-regulation post-NAC was seen in a similar proportion of cases (23/39, 59%) to down-regulation (16/39, 41%) and there was no significant difference between pre- and post-NAC levels.

### Correlations between Pgp, MRP1 and BCRP and with Clinico-pathological Features

Next, we examined whether pre-NAC expression, post-NAC expression or the change in expression after treatment (post-NAC level minus pre-NAC level) for the three markers correlated with each other. Spearman’s rho analyses were performed (Table S1). No significant correlations were found between Pgp and MRP1 or MRP1 and BCRP suggesting that both tumour expression levels and the influence of NAC are independent for these pairs of xenobiotic transporters. Interestingly, a negative and moderately strong correlation was seen between Pgp pre-NAC levels and the change in BCRP expression (rho coefficient −0.5; p = 0.01), meaning that tumours with low initial levels of Pgp were more likely to show larger increases in BCRP expression after NAC. This could suggest some degree of cross-talk between Pgp and BCRP regulation and/or function. As would be expected, for each marker pre- and post-NAC levels were often significantly related to the change in the level for that marker. We also examined whether pre-NAC expression, post-NAC expression or the change in expression after treatment for any of the three markers correlated with patient or tumour characteristics. Parameters tested were patient age at diagnosis, the grade, stage, estrogen receptor status, and her2 status of tumours at diagnosis, and the tumour stage and presence of lymphovascular invasion or axillary metastases as assessed from resection pathology. Spearman’s rho analyses were performed (Table S2). The only significant correlation revealed was a positive and moderately strong correlation between pre-NAC Pgp expression and patient age (rho coefficient 0.41; p = 0.005).

### Pgp, MRP1 and BCRP do not Correlate with Tumour Response to Treatment, but Post-NAC BCRP Correlates with Disease-free Survival

In order to assess the relevance of these markers to clinical outcome, we examined whether their expression or their change in expression correlated with measures of the tumour response to NAC, or with patient survival. With respect to NAC response, we used measures available from data associated with routine clinical assessment of these patients, namely occurrence of complete pathological response, change in either tumour size or stage, and response as assessed by magnetic resonance imaging. First, we assessed whether pre-NAC expression in the six patients who achieved complete pathological responses (pCR; no residual tumour cells identified in the resection) significantly differed from expression in the 39 who did not. There were no significant differences for MRP1 or BCRP. Surprisingly, Pgp expression was significantly higher in the patients with pCR than in those without (Mann-Whitney U test: p = 0.013). Next, Spearman’s rho analyses were performed to examine correlations with the change in tumour size, as assessed by comparison of magnetic resonance imaging scans before NAC with resection pathology, the change in tumour stage, and response as assessed clinically by magnetic resonance imaging during treatment; no significant correlations were found (Table S3).

**Figure 3:**
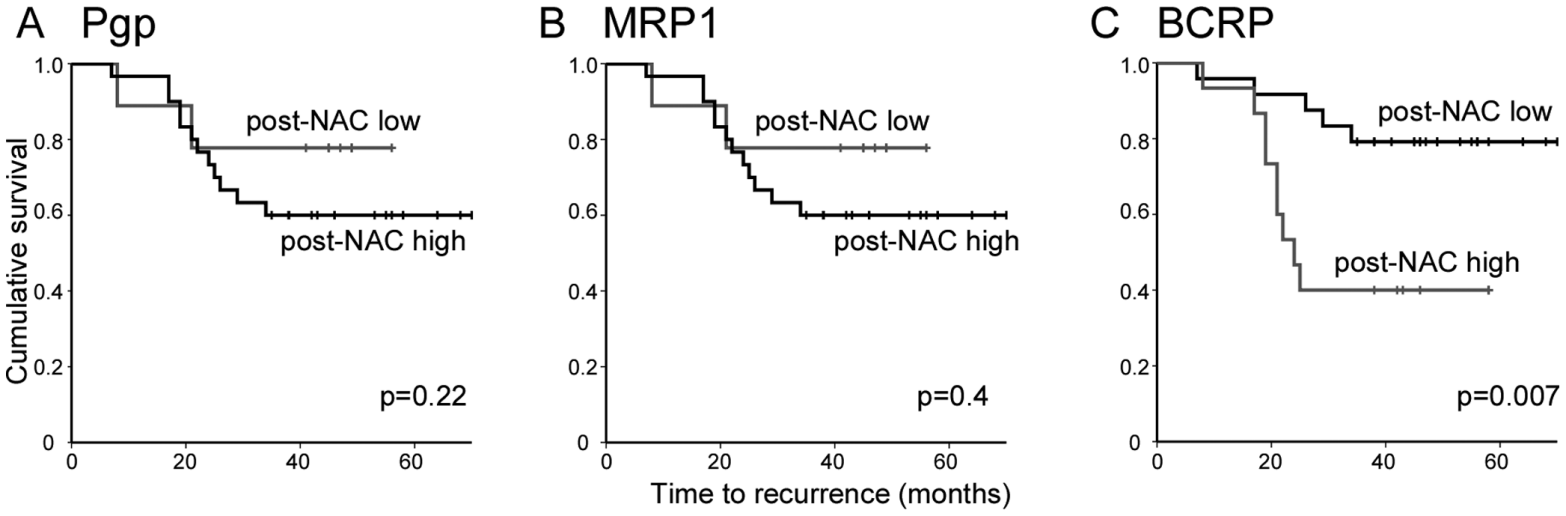
Post-NAC expression of BCRP predicts disease-free survival. Kaplan–Meier survival analyses for disease-free survival in patient groups with tumours with high or low post-NAC expression levels of Pgp (A), MRP1 (B) or BCRP (C). Cut-off used to dichotomise expression into low and high groups (Pgp: 90; MRP1∶21; BCRP: 47) were determined by ROC curve analyses (Fig. S1).

Next, Kaplan–Meier survival analyses were performed to determine differential survival with respect to pre-NAC or post-NAC expressions. We used Receiver Operating Characteristic (ROC) curve analyses to dichotomise expression scores into high and low expression groups giving the highest combined sensitivity and specificity for the end point of disease-free survival (DFS) (Fig. S1). Survival analyses revealed no significant relationships pre-NAC expression of the three markers and DFS (Fig. S2). Similarly, post-NAC expression of Pgp or MRP1 was not significantly related to DFS (Fig. 3A and B). However, post-NAC expression of BCRP was significantly related to DFS (Fig. 3C; Log rank: p = 0.007), with high BCRP expression indicative of poor DFS. For example, 5-year survival of patients showing high post-NAC BCRP expression was 40%, as compared to 80% survival in those showing low expression. Multivariate Cox regression analysis was performed taking into account BCRP expression and standard prognostic factors (tumour grade, receptor status, axillary metastasis, tumour stage pre-NAC, and lymphovascular invasion). BCRP expression proved to be the only significant factor, with high BCRP associated with a hazard ratio of 4.04 (95% confidence interval, 1.3–12.2; p = 0.013). Finally, Kaplan–Meier survival analyses were performed to determine differential survival with respect to the change in expression after treatment for each of the three markers. Change in expression after treatment was dichotomised as either up- or down-regulation. Analyses revealed no significant relationships with DFS (Fig. S3), although the analysis of MRP1 was limited by the small size of the down-regulation group, which contained only three individuals all of whom did not have recurrences.

## Discussion

There is a clear rationale for an expectation that high tumoural expression of the xenobiotic transporter pumps Pgp, MRP1 or BCRP might associate with poor chemotherapy response, since each is capable of removing chemotherapeutics from inside cells and thereby potentially conferring some degree of drug resistance [2]. Accordingly, the hypothesis that expression levels in resected breast tumours predict survival after adjuvant chemotherapy has frequently been tested, at least for Pgp and MRP1 [4]. However, expression of Pgp and MRP1 can be induced by chemotherapy, and it may be that post-chemotherapy expression levels in the neoadjuvant setting are more informative in terms of predicting survival than pre-chemotherapy levels. We have analysed expression of Pgp, MRP1 and BCRP within breast tumours using immunohistochemistry on matched pre-NAC biopsies and post-NAC resection samples and we have tested whether expression of either predicts NAC response or survival in breast cancer patients. A small number of other studies have also used this strategy to test the relevance of various xenobiotic transporters [7], [23], [28], although none have examined all three markers tested in our work. Our methodology, however is distinct, in that we have made rigorous efforts to ensure that expression assessments made in our matched pairs of biopsy and resection samples were as comparable as possible by analysing paired samples on single slides, thus treating them identically, and by using objective computer-aided scoring. This has allowed us to score expressions as continuous variables and thereby make more accurate assessments of relative expression between matched samples and between different tumours, and to select appropriate cut-offs for survival analyses using objective criteria. Also, we have been able to quantify changes in expression of each marker after NAC, rather than simply determining changes in positivity/negativity [23].

We confirmed previous findings that Pgp and MRP1 are significantly up-regulated after NAC [7], [29], but we found neither pre-NAC levels, post-NAC levels or the change in expression to predict NAC response or survival. For MRP1, these findings differ from previously published work in which pre-NAC MRP1 levels significantly predicted survival [7]. Interestingly, in this previous study MRP1 was detected at high levels in some tumours pre-NAC and up-regulation post-NAC occurred in 57% of cases, while we find uniformly low expression pre-NAC and much more frequent up-regulation (92%). These differences may relate to differences in the cohorts or the methodology. With respect to BCRP, we find that pre-NAC levels have no predictive value and that BCRP levels change widely after NAC. These variable induced-changes are evidently important, however, since post-NAC levels were strongly associated with disease-free survival (Fig. 3C). It is interesting to note that considerable effort has been devoted to development of inhibitors of Pgp and BCRP in the hope that their use may improve chemotherapy efficacy [30]. Our data show that both Pgp and BCRP can be induced by NAC in breast cancers and, at least for BCRP, it is only the post-NAC levels that are relevant in terms of outcome. The lack of prognostic correlation with pre-NAC BCRP levels may explain why BCRP levels did not correlate with NAC response, which could only be influenced by these pre-NAC BCRP levels and the dynamic and variable changes in expression induced by the treatment but not by the eventual post-NAC expression levels. Very little is known about the mechanisms that drive the changes in BCRP expression seen in this context, although diverse regulators including estrogen receptor alpha, hypoxia inducible factor 1 alpha, and peroxisome proliferator-activated receptor are known to modulate BCRP transcription under some circumstances [13]. We conclude that BCRP expression levels post-NAC, but not pre-NAC, offer independent prognostic insights and could be used to aid assignment of adjuvant therapies, although future higher powered studies would add weight to this conclusion.

Two further findings merit some discussion. First, we found tumours with low pre-NAC Pgp levels to show larger increases in BCRP expression after NAC. A superficial explanation for this is that Pgp activity in high Pgp tumours successfully reduced intracellular levels of the chemotherapy drugs, and therefore reduced the stimulus for BCRP up-regulation. However, this appears unlikely since MRP1 up-regulation still occurs. An alterative interpretation is that regulatory cross-talk between transporters allows tumours with initially low Pgp expression to respond to NAC with up-regulation of BCRP. This regulatory complexity has been hinted at previously [31] and may have clinical importance as it potentially suggests that successful therapeutic targeting of Pgp may induce compensatory BCRP up-regulation. Secondly, we found a positive correlation between pre-NAC Pgp expression and patient age. There is only a very limited and entirely conflicting literature relating to this point. Previous studies show either no correlation between Pgp and age [32] or a negative correlation [33], although it is worth highlighting the potential confounding factor that, as with much literature on Pgp, these studies use assays to quantify Pgp that differ from ours and from each other.

## Supporting Information

Figure S1 **ROC curve analyses to select cut-off values to dichotomise pre-NAC or post-NAC expression scores for the end point of disease-free survival (DFS).** Plots show the sensitivity+specificity (y-axis) achieved at different cut-off histoscores (x-axis). Cut-off values giving the highest combined sensitivity and specificity were selected. Pre-NAC: Pgp 55; MRP1 4; BCRP 1. Post-NAC: Pgp 90; MRP1 21; BCRP 47.
(TIF)

Figure S2 **Pre-NAC expression of Pgp, MRP1 or BCRP does not predict disease-free survival.** Kaplan–Meier survival analyses for disease-free survival in patient groups with tumours with high or low pre-NAC expression levels of Pgp (A), MRP1 (B) or BCRP (C). Cut-off used to dichotomise expression into low and high groups (Pgp: 55; MRP1∶4; BCRP: 1) were determined by ROC curve analyses (Fig. S1).
(TIF)

Figure S3 **The change in expression of Pgp, MRP1 or BCRP induced by NAC does not predict disease-free survival.** Kaplan–Meier survival analyses for disease-free survival in patient groups with tumours that show up- or down-regulation of Pgp (A), MRP1 (B) or BCRP (C) after NAC.
(TIF)

Table S1 **Spearman’s correlation coefficients demonstrating relationships between expression pre-NAC or post-NAC, or change in expression (Δ) for Pgp, MRP1 and BCRP.** * denotes significance of p<0.05, while bold denotes significance of p<0.01.
(DOCX)

Table S2 **Spearman’s correlation coefficients demonstrating relationships between expression pre-NAC or post-NAC, or change in expression (Δ) for Pgp, MRP1 and BCRP with patient or tumour factors.** * denotes significance of p<0.05, while bold denotes significance of p<0.01.
(DOCX)

Table S3 **Spearman’s correlation coefficients demonstrating relationships between expression pre-NAC or post-NAC, or change in expression (Δ) for Pgp, MRP1 and BCRP with tumour response.** * denotes significance of p<0.05, while bold denotes significance of p<0.01.
(DOCX)
