# Supplementary figures and images for: Neoadjuvant Chemotherapy Induces Expression Levels of Breast Cancer Resistance Protein That Predict Disease-Free Survival in Breast Cancer
Source: PLoS One. 2013 May 2;8(5):e62766. doi: 10.1371/journal.pone.0062766 (PMC3642197; doi:10.1371/journal.pone.0062766)

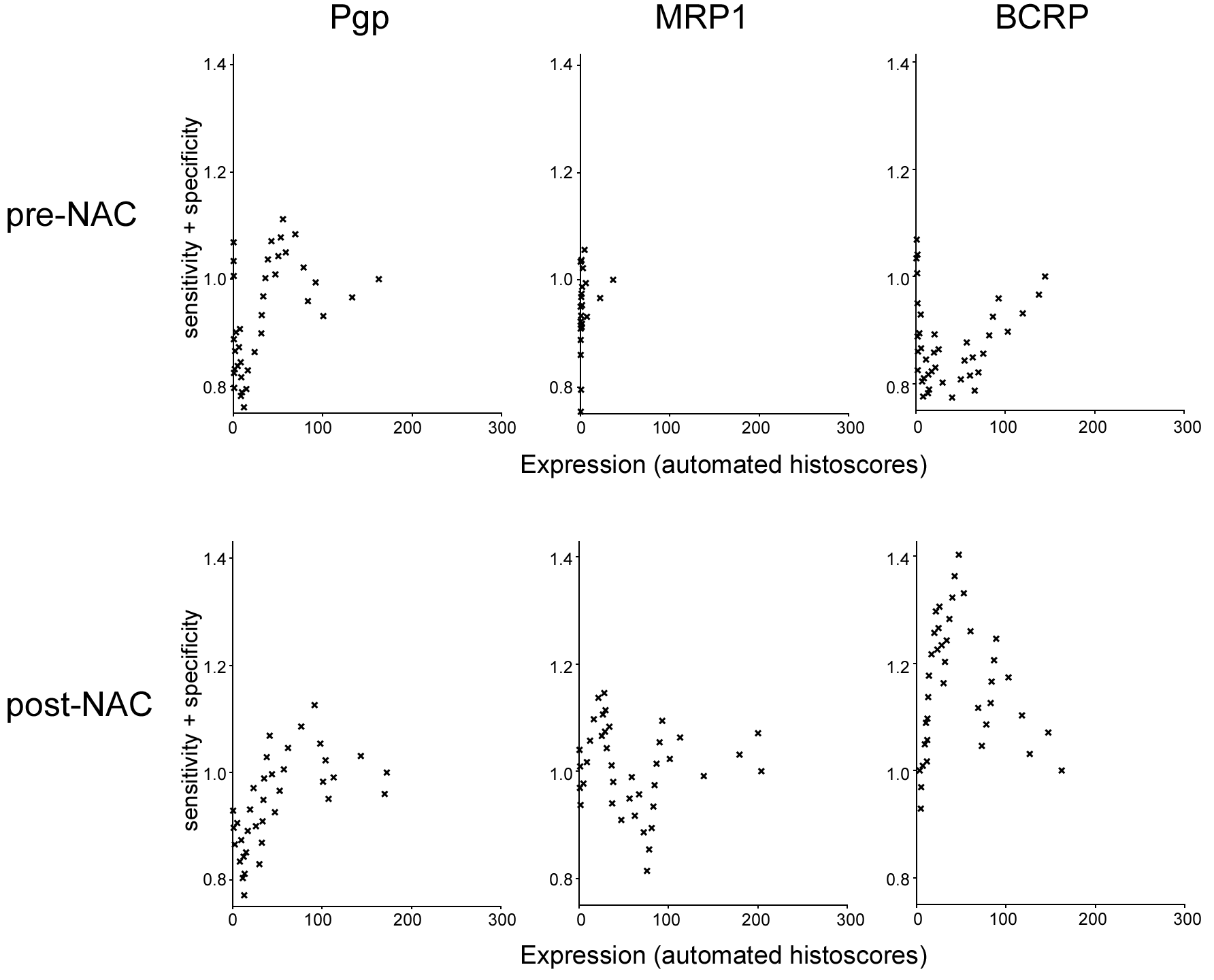

Supplement: Figure S1 — ROC curve analyses to select cut-off values to dichotomise pre-NAC or post-NAC expression scores for the end point of disease-free survival (DFS). Plots show the sensitivity+specificity (y-axis) achieved at different cut-off histoscores (x-axis). Cut-off values giving the highest combined sensitivity and specificity were selected. Pre-NAC: Pgp 55; MRP1 4; BCRP 1. Post-NAC: Pgp 90; MRP1 21; BCRP 47. (TIF) [file pone.0062766.s001.tif]

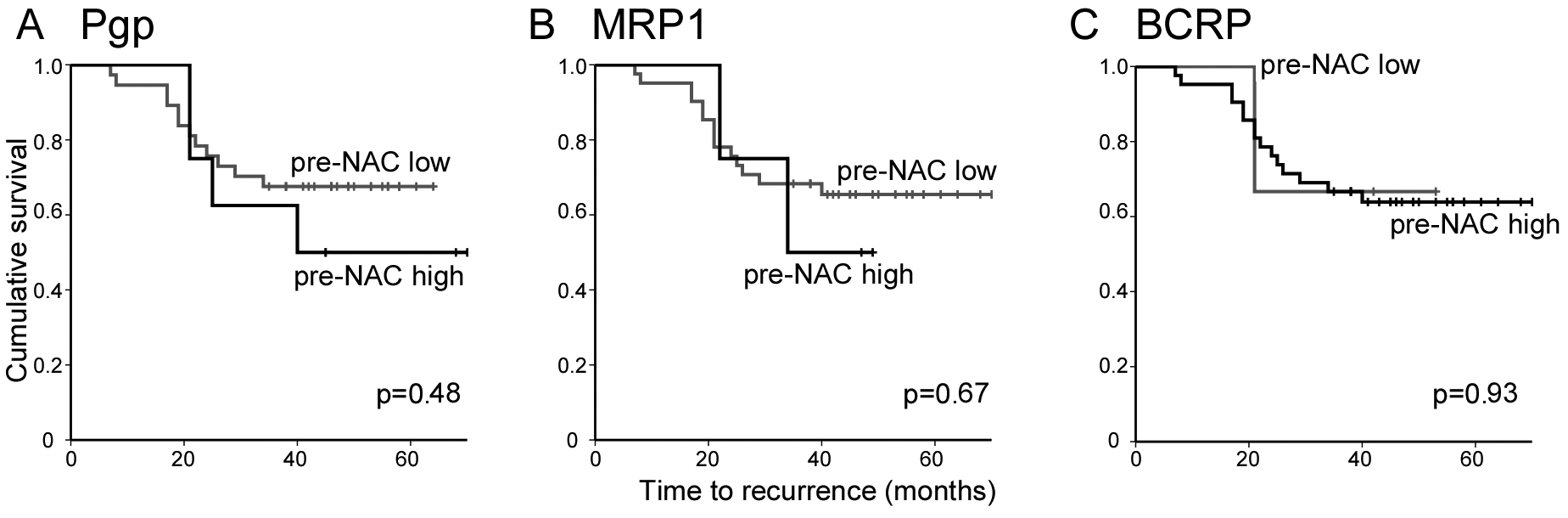

Supplement: Figure S2 — Pre-NAC expression of Pgp, MRP1 or BCRP does not predict disease-free survival. Kaplan–Meier survival analyses for disease-free survival in patient groups with tumours with high or low pre-NAC expression levels of Pgp (A), MRP1 (B) or BCRP (C). Cut-off used to dichotomise expression into low and high groups (Pgp: 55; MRP1∶4; BCRP: 1) were determined by ROC curve analyses (Fig. S1). (TIF) [file pone.0062766.s002.tif]

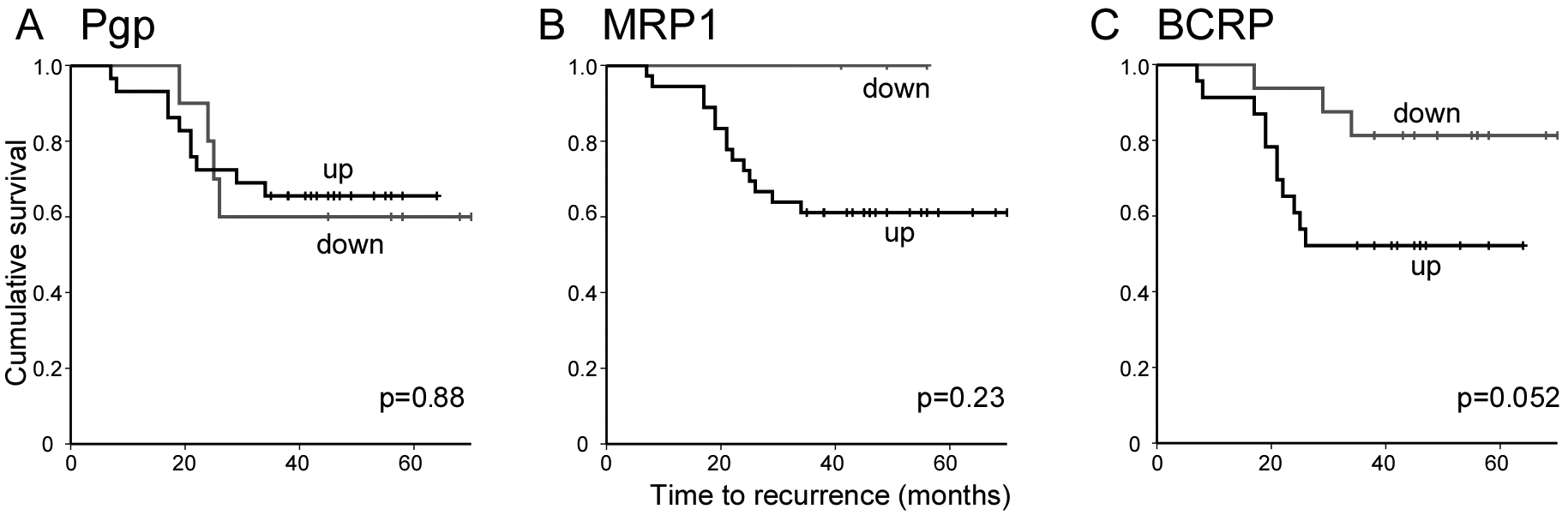

Supplement: Figure S3 — The change in expression of Pgp, MRP1 or BCRP induced by NAC does not predict disease-free survival. Kaplan–Meier survival analyses for disease-free survival in patient groups with tumours that show up- or down-regulation of Pgp (A), MRP1 (B) or BCRP (C) after NAC. (TIF) [file pone.0062766.s003.tif]
