# Supplementary material for: Neoadjuvant Chemotherapy Induces Expression Levels of Breast Cancer Resistance Protein That Predict Disease-Free Survival in Breast Cancer
Source: PLoS One. 2013 May 2;8(5):e62766. doi: 10.1371/journal.pone.0062766 (PMC3642197; doi:10.1371/journal.pone.0062766)
Supplement: Table S1 — Spearman’s correlation coefficients demonstrating relationships between expression pre-NAC or post-NAC, or change in expression (Δ) for Pgp, MRP1 and BCRP. * denotes significance of p<0.05, while bold denotes significance of p<0.01. (DOCX) [file pone.0062766.s004.docx]

|  | Pgp pre | Pgp post | Pgp ∆ | MRP1  pre | MRP1 post | MRP1 ∆ | BCRP pre | BCR post |
| --- | --- | --- | --- | --- | --- | --- | --- | --- |
| Pgp pre | - |  |  |  |  |  |  |  |
| Pgp post | **0.44** | - |  |  |  |  |  |  |
| Pgp ∆ | -0.15 | **0.75** | - |  |  |  |  |  |
| MRP1 pre | 0.09 | 0.07 | 0.11 | - |  |  |  |  |
| MRP1 post | -0.11 | -0.29 | -0.15 | -0.2 | - |  |  |  |
| MRP1 ∆ | -0.12 | -0.28 | -0.28 | -0.25 | **0.99** | - |  |  |
| BCRP pre | 0.27 | 0.07 | -0.13 | 0.24 | -0.03 | -0.05 | - |  |
| BCRP post | -0.34* | -0.16 | 0.05 | -0.17 | 0.14 | 0.14 | -0.12 | - |
| BCRP ∆ | **-0.5** | -0.15 | 0.16 | -0.15 | 0.12 | 0.13 | **-0.77** | **0.63** |

**Table S1**
