# Supplementary material for: Neoadjuvant Chemotherapy Induces Expression Levels of Breast Cancer Resistance Protein That Predict Disease-Free Survival in Breast Cancer
Source: PLoS One. 2013 May 2;8(5):e62766. doi: 10.1371/journal.pone.0062766 (PMC3642197; doi:10.1371/journal.pone.0062766)
Supplement: Table S2 — Spearman’s correlation coefficients demonstrating relationships between expression pre-NAC or post-NAC, or change in expression (Δ) for Pgp, MRP1 and BCRP with patient or tumour factors. * denotes significance of p<0.05, while bold denotes significance of p<0.01. (DOCX) [file pone.0062766.s005.docx]

|  | Pgp pre | Pgp post | Pgp ∆ | MRP1 pre | MRP1 post | MRP1 ∆ | BCRP pre | BCRP post | BCRP ∆ |
| --- | --- | --- | --- | --- | --- | --- | --- | --- | --- |
| Age at diagnosis | **0.41** | 0.31 | 0.04 | 0.1 | -0.13 | -0.13 | 0.08 | -0.18 | -0.33* |
|  |  |  |  |  |  |  |  |  |  |
| Tumour factors  determined pre-NAC: |  |  |  |  |  |  |  |  |  |
| Grade | 0.19 | -0.16 | -0.3 | 0.16 | -0.02 | 0.01 | -0.09 | 0.04 | 0.04 |
| T Stage (MRI) | 0.06 | -0.09 | -0.13 | -0.14 | 0.29 | 0.27 | -0.26 | -0.16 | 0.04 |
| ER status | -0.08 | -0.17 | -0.12 | 0.24 | 0.13 | 0.11 | 0.18 | -0.07 | -0.06 |
| Her2 status | -0.03 | 0.21 | 0.33* | -0.18 | 0.01 | 0.01 | 0.01 | -0.02 | 0.03 |
|  |  |  |  |  |  |  |  |  |  |
| Tumour factors determined post-NAC: |  |  |  |  |  |  |  |  |  |
| T stage (resection pathology) | -0.36* | -0.15 | 0.04 | 0.22 | 0.14 | 0.1 | 0.14 | 0.05 | 0.05 |
| Lymphovascular invasion | -0.35 | -0.1 | 0.04 | 0.04 | 0.23 | 0.24 | 0.17 | -0.38* | -0.23 |
| Axillary metastasis | -0.07 | 0.19 | 0.3 | 0.38* | -0.1 | -0.11 | 0.36* | -0.08 | -0.26 |

**Table S2**
