# Supplementary material for: Neoadjuvant Chemotherapy Induces Expression Levels of Breast Cancer Resistance Protein That Predict Disease-Free Survival in Breast Cancer
Source: PLoS One. 2013 May 2;8(5):e62766. doi: 10.1371/journal.pone.0062766 (PMC3642197; doi:10.1371/journal.pone.0062766)
Supplement: Table S3 — Spearman’s correlation coefficients demonstrating relationships between expression pre-NAC or post-NAC, or change in expression (Δ) for Pgp, MRP1 and BCRP with tumour response. * denotes significance of p<0.05, while bold denotes significance of p<0.01. (DOCX) [file pone.0062766.s006.docx]

|  | Pgp pre | Pgp post | Pgp ∆ | MRP1 pre | MRP1 post | MRP1 ∆ | BCRP  pre | BCRP post | BCRP ∆ |
| --- | --- | --- | --- | --- | --- | --- | --- | --- | --- |
| ∆ T stage | -0.25 | -0.08 | 0.11 | 0.19 | 0.09 | 0.06 | 0.1 | 0.15 | 0.07 |
| MRI response | 0.2 | 0.07 | 0.004 | -0.28 | 0.04 | 0.08 | 0.03 | -0.09 | -0.2 |
| ∆ in tumour size | -0.25 | -0.16 | -0.02 | -0.00 | 0.15 | 0.16 | 0.17 | 0.37* | 0.21 |

**Table S3**
